# Characterizing the V516M penicillin-binding protein 2 (PBP2) mutation causing zidebactam resistance in *Pseudomonas aeruginosa*

**DOI:** 10.1128/aac.00346-26

**Published:** 2026-06-15

**Authors:** Miquel Àngel Sastre-Femenia, Maria Antonia Gomis-Font, Almudena Fernández-Muñoz, Daniela Salazar-Londoño, Bartolome Moya, Antonio Oliver

**Affiliations:** 1 Department of Microbiology, Hospital Universitari Son Espases, Health Research Institute of the Balearic Islands (IdISBa), CIBERINFEC 219656 , Palma, Spain; University of Fribourg, Fribourg, Switzerland

**Keywords:** resistance mechanisms, *Pseudomonas aeruginosa*, penicillin-binding proteins

## Abstract

The triple action of the diazabicyclooctane (DBO) zidebactam (antibacterial activity [penicillin-binding protein 2 {PBP2} inhibitor], β-lactam enhancement [synergy with PBP3 inhibitors], and β-lactamase inhibition [classes A and C]) provides a promising approach to overcome *Pseudomonas aeruginosa* resistance. However, PBP2 mutations (particularly V516M) are associated with resistance. Thus, we characterized the effect of PBP2_V516M_ mutation on the activity and PBP-binding affinities (50% inhibitory concentrations [IC_50_s]) of a panel of β-lactams and DBOs using a PBP2_V516M_ mutant and combinations with *dacB*, *mexR*, *mexZ*, or *oprM* knockout mutations. V516M dramatically increased zidebactam minimum inhibitory concentration and PBP2 IC_50_ and determined specific patterns of β-lactams and DBO susceptibilities and PBP-binding profiles. Nacubactam showed cross-resistance with zidebactam but showed a much lower basal binding affinity against PBP2, explaining its lower intrinsic antipseudomonal activity. Conversely, durlobactam did not show cross-resistance with zidebactam, consistent with its primary target being PBP1b and not PBP2; the low intrinsic activity of durlobactam was related to efflux. Likewise, PBP2 binding of mecillinam was similar to zidebactam, but its lower antipseudomonal activity was related to the higher contribution of efflux (MexAB-OprM). In contrast, imipenem was barely affected by the PBP2_V516M_ mutation. Zidebactam resistance did not affect its activity as an AmpC inhibitor, restoring cefepime susceptibility in the *dacB* mutant. The PBP2_V516M_ mutation was associated with cell morphology changes, but it did not affect growth rate or virulence. Antibiotic resistance and PBP IC_50_ profiling of PBP2_V516M_ mutation help to understand the impact of target modification resistance mechanisms on existing DBOs and provide clues for guiding the development of novel derivatives.

## INTRODUCTION

*Pseudomonas aeruginosa*, a ubiquitous opportunistic pathogen considered one of the paradigms of antimicrobial resistance, is among the main causes of hospital-acquired and chronic infections associated with high morbidity and mortality (1). Moreover, carbapenem-resistant *P. aeruginosa* was included in the top (critical) priority in the first WHO list for the need for research and development of new antibiotics (2) and is still considered a high priority in the 2024 updated document (3).

This growing threat results from the interplay of the extraordinary capacity of *P. aeruginosa* for developing resistance through chromosomal mutations, the increasing prevalence of transferable resistance determinants (such as carbapenemases), and the global dissemination of multidrug-resistant (MDR)/extensively drug-resistant (XDR) epidemic lineages (the so-called high-risk clones) (4). A number of novel β-lactams and combinations with β-lactamase inhibitors, such as ceftolozane/tazobactam, ceftazidime/avibactam, imipenem/relebactam, or cefiderocol, have been introduced over the last decade, mitigating the problem to some extent, but emerging resistance mechanisms are being reported (5).

Zidebactam was the first described gram-negative β-lactam enhancer belonging to the bicyclo-acyl hydrazide (BCH) series with stand-alone anti-pseudomonal activity (6). Moreover, the combination of cefepime and zidebactam is currently under clinical development for infections caused by MDR/XDR gram-negative bacteria, including *P. aeruginosa*. Although derived from a diazabicyclooctane (DBO) scaffold like avibactam or relebactam, BCHs were designed with the objective of augmenting penicillin-binding protein 2 (PBP2) binding as opposed to the conventional approach of optimizing the β-lactamase-inhibitory activity (6). The tri ple action (direct antibacterial activity, β-lactam enhancer activity, and class A and C β-lactamase inhibition) provided by zidebactam offers a unique mechanistic opportunity to simultaneously overcome *P. aeruginosa* resistance mechanisms, even concerning MBLs, when combined with cefepime or other PBP3 inhibitor β-lactams (6, 7). However, resistance development to zidebactam and cefepime/zidebactam has been well documented *in vitro* (8). Of particular interest, these experiments have revealed that specific PBP2 mutations are associated with zidebactam resistance, but their characterization is still limited. Among them, the V516M mutation is particularly noteworthy, as it was readily selected upon zidebactam exposure in different independent experiments and in different strain backgrounds (8, 9). Therefore, the objective of this work was to characterize the effect of this mutation on the antipseudomonal activity and PBP binding of a meaningful panel of β-lactams and DBOs, such as nacubactam and durlobactam.

## MATERIALS AND METHODS

### Strains, plasmids, and construction of PAO1 mutants

The complete list of strains and plasmids used or constructed in this work is shown in Table S1. Isogenic mutant PAO1 PBP2_V516M_ was constructed using the allelic exchange method (10). Briefly, upstream and downstream primers flanking the *pbpA* gene (Table S2) were designed to amplify a 2.6 kb genomic region to promote efficient homologous recombination. The fragment was amplified by PCR using a genomic DNA template harboring the target mutation derived from an *in vitro* evolved strain (8). Following this, the PCR product was digested with EcoRI and HindIII, ligated to pEX18Gm^R^, and transformed into *Escherichia coli* XL_1_ Blue. Transformants were selected on LB agar plates containing gentamicin (5 mg/L), X-gal, and IPTG, and positive colonies were subsequently checked by Sanger sequencing to ensure that no additional mutation had been introduced into the *pbpA* cloned fragment. Verified plasmids were then transformed into the *E. coli* S17.1 λ-pir strain. Isogenic mutants were generated by conjugation, selecting the resultant merodiploids on cefotaxime (1 mg/L) and gentamicin (30 mg/L) LB agar plates. Double recombinants were counterselected on LB no-sodium sucrose agar plates after incubation of merodiploids on BHI broth. Resulting mutants were confirmed by Sanger and whole genome sequencing to ensure no relevant mutations were introduced for subsequent experiments. Knockout mutants in *dacB* (PBP4), *mexR*, *oprM*, or *mexZ* of PAO1 PBP2_V516M_ were constructed using the *Cre*-*lox* system for gene deletion and antibiotic resistance marker recycling following previously described protocols (11). Previously transformed *E. coli* S17.1 λ-pir strains with pEX100Tlink containing the flanking sequence of the genes disrupted with the *lox-flanked* gentamicin resistance cassette (*aac1*) were used. The knockout mutants were generated by conjugation followed by selection of double recombinants using sucrose, cefotaxime (1 mg/mL), and gentamicin (30 mg/L) LB agar plates and checked by PCR. To eliminate the gentamicin resistance cassette, plasmid pCM157 was electroporated into the different mutants, and transformants were selected in tetracycline (250 mg/L) LB agar plates. One transformant for each mutant was grown overnight in LB broth in order to allow expression of the *cre* recombinase, and then plasmid pCM157 was cured by successive passages on LB broth. Finally, selected colonies were then screened for susceptibility to tetracycline (250 mg/L) and gentamicin (30 mg/L) and checked by PCR amplification to verify the cassette elimination.

### Susceptibility testing

Minimum inhibitory concentrations (MICs) of cefepime, imipenem, zidebactam, cefepime/zidebactam (1:1 ratio), nacubactam, durlobactam, mecillinam, relebactam, and avibactam were determined by in-house broth microdilution according to EUCAST guidelines (http://eucast.org) using cation-adjusted Mueller-Hinton broth in triplicate experiments. Inhibitors were tested from 1 to 512 mg/L, and imipenem, cefepime, and its combination with zidebactam were tested from 0.12 to 64 mg/L. MIC values were represented as a range when the three replicates did not show identical results.

### Growth rates

To assess the growth rates of PAO1 and its mutant PBP2_V516M_, optical density at 600 nm (OD_600_) and CFU per milliliter were determined over a 24-h period. Overnight LB broth cultures of PAO1 and PAO1 PBP2_V516M_ were diluted to a final OD_600_ of 0.05 in fresh medium and incubated at 37°C and 180 rpm. Growth was monitored by measuring OD_600_ at 1-h intervals from 0 to 8 and 24 h, and serial dilutions were plated at 0, 1, 4, 8, and 24 h to determine the CFU per milliliter. In addition, live-dead staining was conducted to observe the bacterial morphology at exponential growth. The LIVE/DEAD BacLight bacterial viability kit was used following the manufacturer’s instructions. All experiments were performed in triplicate.

### Gene expression

The expression of the gene *pbpA* encoding the PBP2 was determined from the late log-phase (OD_600_ of 1) LB cultures at 37°C and 180 rpm by real-time RT-PCR with a Bio-Rad instrument (CFX Connect Real-Time System), as previously described in protocols (12). The primers listed in Table S2 were used for amplification of *pbpA* and *rpsL* (used as a reference to normalize the relative amounts of mRNA).

### PBP-binding assays

Membrane preparations containing the PBPs of PAO1 and the PAO1 PBP2_V516M_ mutant were obtained following previously described protocols with slight modifications (13, 14). Briefly, 1 L of late-log-phase (OD_600_ of 1) LB cultures was collected by centrifugation, washed, and resuspended in Dulbecco’s phosphate-buffered saline (PBS). Cells were then sonicated and centrifuged at 3,220 RCF for 30 min. Membranes containing the PBPs were isolated through one step of ultracentrifugation at 150,000 RCF at 4°C for 30 min. PBPs were then incubated for 30 min at 37°C in the presence of increasing concentrations of imipenem, zidebactam, mecillinam, nacubactam, and durlobactam. Afterwards, PBPs were labeled with a 25 µM concentration of BOCILLIN FL fluorescent penicillin and separated through 10% SDS-polyacrylamide gel electrophoresis (SDS-PAGE). Finally, PBPs were visualized using Odyssey M, and 50% inhibitory concentrations (IC_50_s) for the different PBPs were determined from two independent membrane preparations by quantification through the Odyssey LI-COR Imaging System, calculating the mean value ± standard deviation (SD). The range of concentrations tested was 0.0156–2 mg/L, and when PBP2 was not inhibited at 2 mg/L, the range was expanded to 1–128 mg/L in a subsequent experiment. Stand-alone (without competitor antibiotic) BOCILLIN FL-labeled PBP profiles of PAO1 and the PBP2_V516M_ mutant were also compared, and the relative amount of PBP2 (band intensity) was quantified by adjusting the total loaded protein content and normalizing to the PBP3 band intensity in each strain.

### *Caenorhabditis elegans* killing assay

Bacterial killing of *C. elegans* was performed as described previously (15). Briefly, a fresh culture of PAO1 and PAO1 PBP2_V516M_ was layered onto a 55 mm diameter plate containing potato dextrose agar and incubated at 37°C for 24 h to form bacterial lawns. Five worms per plate were then placed on top and incubated at 24°C, numbering the living worms at 0, 24 , 48, 72, and 168 h. Three independent experiments were performed, and mean ± SD values were recorded. The *E. coli* strain OP50 served as a non-pathogenic control. *C. elegans* virulence scores 1–5 were assigned following previously established categories (15).

### *Galleria mellonella* infection model

The wax moth *G. mellonella* was used as the infection model, following previously described protocols (16, 17). Exponentially growing cultures of PAO1 and its mutant, PBP2_V516M_, were pelleted, washed, and resuspended in PBS. Serial dilutions were made in PBS and injected into individual *G. mellonella* larvae using Hamilton syringes (10 µL) via the hindmost left proleg. Ten larvae were injected for each dilution and scored as live or dead after 20 h at 37°C. Final numbers of injected bacteria were verified by plating serial dilutions and colony counts. Three independent experiments were performed, and the lethal dose (LD_50_) ± SD was determined using R software, version 4.4.1. In all cases, 10 larvae were inoculated with 10 µL of PBS as control.

## RESULTS

### Impact of PBP2_V516M_ mutation on antimicrobial susceptibility

As shown in Table 1, the PBP2_V516M_ mutation led to a dramatic increase in zidebactam MICs, from 4 to 128 µg/mL. MICs of avibactam and relebactam were already >256 µg/mL in parent PAO1 and thus were not further tested. However, durlobactam and nacubactam did show some activity against wild-type PAO1 and thus were further tested in the PBP2_V516M_ mutant. Remarkably, opposite results were obtained for both DBOs: the MIC of nacubactam was significantly increased in the PBP2 mutant, while that of durlobactam remained unchanged. Regarding β-lactams, the activity of cefepime was not significantly affected, but the MICs of the cefepime/zidebactam combination were slightly increased. The MICs of imipenem were also only slightly increased (one twofold dilution), whereas the MICs of mecillinam were notably enhanced. The effect on resistance of the combination of the PBP2_V516M_ mutation with those mutations leading to AmpC overexpression (*dacB*/PBP4) and efflux pump overexpression (*mexR* [MexAB-OprM] or *mexZ* [MexXY-OprM]) or inactivation (*oprM*) was also assessed. As shown in Table 1 zidebactam restored cefepime MICs in the PBP2 _V516M_-*dacB* mutant, indicating that zidebactam resistance driven by the PBP2 mutation did not alter its activity as an AmpC inhibitor. Conversely, MICs of zidebactam alone or when combined with cefepime were slightly increased in the efflux pump overexpressing strains and decreased in those defective. The MICs of durlobactam, but not those of nacubactam, were also modulated by efflux pump expression. Conversely, the MICs of cefepime and mecillinam, unlike those of imipenem, were dramatically reduced in the efflux pump defective *oprM* mutant.

**TABLE 1:** MICs of isogenic derivative mutants of PAO1^*a*^

	MIC (mg/L)						
Strain	FEP	ZID	FEP/ZID (1:1)	IMP	NAC	DUR	MEC
WT	2	4	1	0.5	128	64	128
PBP2_V516M_	2–4	128	2–4	1	>512	64	>512
WT Δ*oprM*	≤0.12	1–2	≤0.12	0.5	128	NT	2
PBP2_V516M_ Δ*oprM*	≤0.12	64	≤0.12	1	>512	8–16	32
WT Δ*mexR*	4–8	4	4–8	0.5	128	NT	>512
PBP2_V516M_ Δ*mexR*	4–8	256	4–8	1	>512	256	>512
WT Δ*dacB*	16	4	2	0.5	128	NT	256–512
PBP2_V516M_ Δ*dacB*	16	256	2–4	1	>512	64	>512
WT Δ*mexZ*	4	4	2	0.5	128	NT	256–512
PBP2_V516M_ Δ*mexZ*	4	256	4	1	>512	64	>512

^*a*^ MIC, minimum inhibitory concentration; NT, not tested.

### Impact of PBP2_V516M_ mutation on PBP-binding affinities

**Fig 1:**
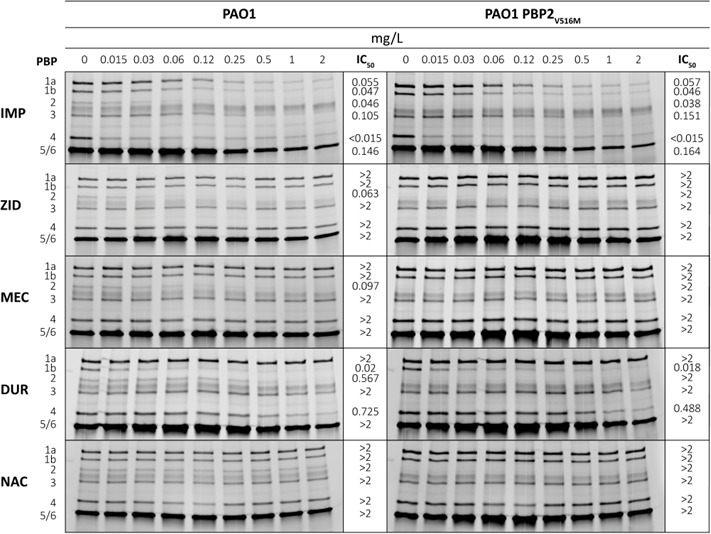
Representative SDS-PAGE images and corresponding PBP-binding affinity (IC_50_) values of imipenem, zidebactam, mecillinam, durlobactam, and nacubactam for wild-type PAO1 and its PBP2_V516M_ mutant.

**Fig 2:**
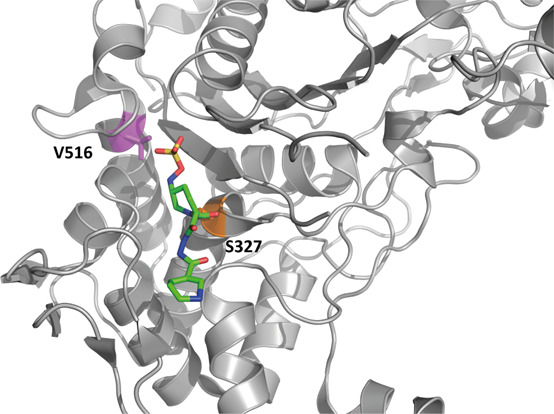
Docking analysis using PyMOL of the interaction of zidebactam and PBP2 with V516 and S327 (catalytic serine) residues highlighted.

Table 2 contains the PBPs IC_50_s for zidebactam and relevant comparators in PAO1 and the PBP2_V516M_ mutant, while Fig. 1 shows the corresponding representative SDS-PAGE images. As shown, the PBP2_V516M_ mutation determined a dramatic increase in the zidebactam IC_50_s of PBP2, from 0.063 to >128 µg/mL (Table 2). The relevance of the V516 residue is also supported by molecular docking results that revealed its interaction with zidebactam in the catalytic center of PBP2 (Fig. 2). As expected, zidebactam failed to bind to any other PBP, neither in wild-type PAO1 nor in the PBP2_V516M_ mutant. Very similar results were obtained for mecillinam (from 0.097 to >128), confirming shared specific PBP2 binding of both agents. As shown in Table 1, the reduced antipseudomonal activity of mecillinam as compared with zidebactam is thus more related to efflux pump expression rather than PBP2 target binding. As expected, imipenem bound all PBPs efficiently, but no significant differences were observed for the PBP2_V516M_ mutant as compared with PAO1, with PBP2 IC_50_s of 0.046 µg/mL and 0.038 µg/mL, respectively. Regarding the other DBOs, nacubactam behaved similarly to zidebactam, showing specific affinity for PBP2, but with markedly lower potency. Basal PBP IC_50_s of nacubactam in PAO1 were 2.5 µg/mL (roughly 40-fold higher than those of zidebactam) and increased to >128 µg/mL in the PBP2_V516M_ mutant. Thus, the much lower antipseudomonal activity of nacubactam compared with zidebactam related smoothly to the lower PBP2 target affinity. In contrast, durlobactam’s highest affinity in wild-type PAO1 was for PBP1b (0.02 µg/mL), while that of PBP2 was 0.57 µg/mL (intermediate between zidebactam and nacubactam). Moreover, durlobactam PBP2 IC_50_s increased in the PBP2_V516M_ mutant but not as dramatically as for zidebactam or nacubactam. All results together indicate that the antipseudomonal activity of durlobactam relies more on PBP1b inhibition rather than PBP2, explaining the lack of cross-resistance between zidebactam and durlobactam.

**TABLE 2:** PBP-binding affinities (IC_50_) of imipenem, zidebactam, mecillinam, durlobactam, and nacubactam

	IC_50_									
	IMP		ZID		MEC		DUR		NAC	
PBP	PAO1	PBP2_V516M_	PAO1	PBP2_V516M_	PAO1	PBP2_V516M_	PAO1	PBP2_V516M_	PAO1	PBP2_V516M_
1a	0.055 ± 0.001	0.057 ± 0.002	>2	>2	>2	>2	>2	>2	>128	>128
1b	0.047 ± 0.004	0.046 ± 0.004	>2	>2	>2	>2	0.02 ± 0.007	0.0175 ± 0.004	67.2795 ± 18.65	56.8055 ± 2.939
2	0.046 ± 0.011	0.038 ± 0.001	0.063 ± 0.015	>128	0.097 ± 0.006	>128	0.567 ± 0.03	7.997 ± 0.895	2.5105 ± 0.643	>128
3	0.105 ± 0.001	0.151 ± 0.018	>2	>2	>2	>2	>2	>2	>128	>128
4	<0.015	<0.015	>2	>2	>2	>2	0.725 ± 0.13	0.4875 ± 0.18	45.105 ± 16.56	51.087 ± 5.037
5/6	0.146 ± 0.060	0.164 ± 0.008	>2	>2	>2	>2	>2	>2	>128	>128

### Impact of PBP2_V516M_ mutation in cell morphology, growth rate, and virulence

**Fig 3:**
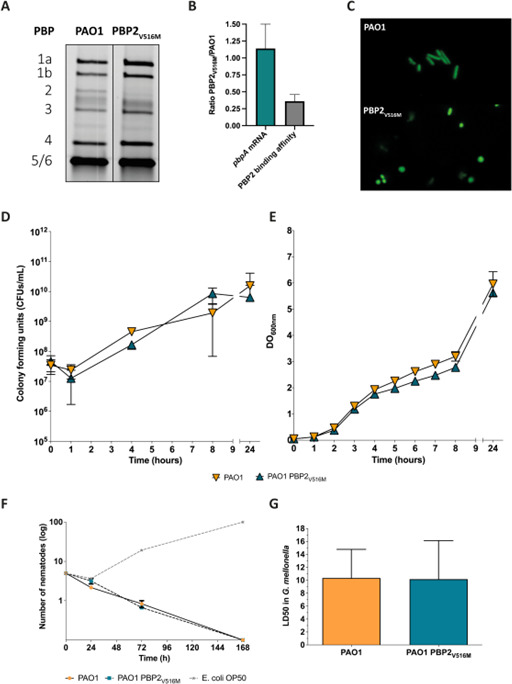
Comparative analysis of PAO1 and its PBP2_V516M_ mutant. (**A**) SDS-PAGE PBP profile (obtained from controls without antibiotic of Fig. 1). (**B**) Relative *pbpA* mRNA expression and PBP2-binding affinity. (**C**) Live-dead staining confocal microscopy images. (**D**) Growth curves in CFU per milliliter. (**E**) Growth curves in OD_600nm_. (**F**) *C. elegans* virulence assay (overtime counts of surviving nematodes). (**G**) *G. mellonella* LD_50_.

Beyond the IC_50_s, PBP-binding profiling of the PBP2_V516M_ mutant revealed a lower (0.36 ± 0.10) PBP2 expression compared to PAO1 (Fig. 3). However, *pbpA* gene expression was not modified. Moreover, WGS analysis failed to reveal any further secondary mutation beyond V516M. Therefore, these findings suggest that the activity of PBP2 is reduced in the PBP2_V516M_ mutant. Indeed, microscopic (live/dead staining) morphology assessment revealed rounder cells supporting a partially defective activity of PBP2 in the PBP2_V516M_ mutant (Fig. 3). Thus, the potential impact of this defect on fitness and virulence was assessed. However, as shown in Fig. 3, neither growth rate nor virulence in two invertebrate models (*G. mellonella* and *C. elegans*) was affected in the PBP2_V516M_ mutant.

## DISCUSSION

Nearly one century after the discovery of penicillin, β-lactams continue to be the front line of our antibacterial arsenal. For decades, however, the development of new β-lactams and β-lactamase inhibitors has barely followed the path of resistance mechanisms developed by gram-negative pathogens. The introduction of DBOs and other non-β-lactam β-lactamase inhibitors, such as the boronates, in the last decade has provided a new life for β-lactams and even extended beyond, opening a new dimension with the development of new non-β-lactam PBP inhibitors (18, 19). One of the first of such promising molecules was zidebactam, currently under clinical development in combination with cefepime for the treatment of MDR gram-negative pathogens.

Indeed, the strong PBP2 inhibition of zidebactam has been demonstrated to confer potent *in vitro* and *in vivo* bactericidal activity when combined with PBP3 inhibitors such as cefepime against a wide range of MDR gram-negative pathogens, including those not only producing β-lactamases inhibited by DBOs (classes A and C) but also challenging MBL-producing *P. aeruginosa*, MBL-producing *Klebsiella pneumoniae*, and OXA carbapenemase-producing *Acinetobacter baumannii* (6, 7, 20, 21).

Understanding the PBP2 targeting activity of zidebactam and potential resistance drivers is thus of major interest. Previous *in vitro* evolution experiments evidenced the selection of specific PBP2 mutations upon zidebactam (and cefepime/zidebactam) exposure (8). Among them, the V516M mutation was consistently selected in independent experiments (8, 9). In line with previous works (8, 9), detailed characterization of this mutation evidenced not only that it confers a dramatic increase in zidebactam MICs and PBP2 IC_5O_s but also that it determines specific profiles of β-lactam and DBO susceptibilities and PBP-binding profiles. Understanding such differential patterns should be helpful for the development of new molecules with enhanced activity and stability against resistance development by PBP2 target mutation. Of particular relevance is the differential impact evidenced for nacubactam and durlobactam. Nacubactam showed high-level cross-resistance with zidebactam through the V516M mutation, but it also showed a much lower basal binding affinity against PBP2, explaining its much lower intrinsic antipseudomonal activity. Conversely, durlobactam did not show cross-resistance with zidebactam through the V516M mutation, consistently with the fact that its primary target was found to be PBP1b and not PBP2. Moreover, the low intrinsic activity of durlobactam against *P. aeruginosa* is found to be, at least partially, related to intrinsic efflux pump (MexAB-OprM) activity. Likewise, our result revealed that the PBP2 target binding of mecillinam and zidebactam is very similar and that the much lower antipseudomonal activity of the former is in fact related to the higher contribution of efflux (MexAB-OprM) to intrinsic resistance. Moreover, our results also evidenced that the acquisition of zidebactam resistance by PBP2 mutation did not affect its activity as a β-lactamase (AmpC) inhibitor, restoring cefepime susceptibility in the *dacB* mutant.

Another relevant finding is that, while the V516M mutation impacted bacterial morphology, generating round cells, it had no major impact on growth rates and virulence, suggesting that it could be eventually selected *in vivo*. Moreover, a recent study suggested that this mutation could increase virulence in a murine pneumonia model (9). However, PBP2 sequence is found to be highly conserved among *P. aeruginosa* clinical isolates (22), and extensive analysis of our large (>500) collections of XDR *P. aeruginosa* genomes (23–25) failed to identify this mutation in any of them. Nevertheless, a close substitution, V517M, was noted in one isolate from a complex set of carbapenemase-producing *P. aeruginosa* strains showing cefepime/zidebactam reduced activity (26). Moreover, variation of PBP2 sequences associated with reduced cefepime/zidebactam susceptibility in carbapenemase-producing *E. coli* from Sweden has also been recently reported (27).

*In vitro* evolution experiments have also revealed that PBP2 mutations can be combined with PBP3 mutations to drive cefepime/zidebactam resistance but are associated with a high fitness cost (8). Moreover, beyond PBP mutations, efflux pumps were also noted to be involved in cefepime/zidebactam resistance development in *in vitro* evolution experiments (8). Indeed, the first and only so far reported case of resistance development to cefepime/zidebactam during treatment of a patient involved efflux pumps and no PBP mutations (28). Thus, the clinical relevance of V516M or other PBP2 mutations still needs to be demonstrated through close monitoring of treated patients. In this regard, PBP3 mutations have already been well documented *in vivo* upon treatment with cephalosporins or meropenem, even if associated with a fitness cost related to reduced cell division (29).

In summary, from a broader perspective, our work, delving into the analysis of PBP-binding profiles and target mutations, provides useful information and determines a step forward in the current scenario of renewed interest in the development of a whole range of different non-β-lactam PBP inhibitors, with or without coupled β-lactamase inhibitory activity, for the treatment of infections by MDR gram-negative pathogens (19, 30–32).

## SUPPLEMENTAL MATERIAL

Supplemental material aac.00346-26-s0001.pdf Tables S1 and S2.

ASM does not own the copyrights to Supplemental Material that may be linked to, or accessed through, an article. The authors have granted ASM a non-exclusive, world-wide license to publish the Supplemental Material files. Please contact the corresponding author directly for reuse.
