# Supplementary material for: Characterizing the V516M penicillin-binding protein 2 (PBP2) mutation causing zidebactam resistance in Pseudomonas aeruginosa
Source: Antimicrob Agents Chemother. 2026 Jun 15;70(8):e00346-26. doi: 10.1128/aac.00346-26 (PMC13435639; doi:10.1128/aac.00346-26)
Supplement: Supplementary file 1 [file aac.00346-26-s0001.pdf]

Table S1. Strains and plasmids used or constructed in this study.

**Strain or plasmid**	**Genotype and relevant characteristic(s)**	**Reference or source**
Strains		
P. aeruginosa		
PAO1	Reference strain completely sequenced	Laboratory collection
PAO1 ΔoprM	PAO1 Δ*oprM::lox*; *oprM* encoding the outer membrane protein component of MexAB-OprM and MexXY-OprM efflux pumps	Mulet X et al 2011
PAO1 ΔmexR	PAO1 Δ*mexR::lox*; *mexR* encoding the negative regulator of MexAB-OprM efflux pump	Mulet X et al 2011
PAO1 ΔdacB	PAO1 Δ*dacB::lox*; *dacB* encoding the nonessential PBP4	Moya B 2009
PAO1 ΔmexZ	PAO1 Δ*dmexZ::lox*; *mexZ* encoding the negative regulator of MexXY-OprM	Torrens G et al 2016
PAO1 $PBP2_{V516M}$	PAO1 carrying mutation V516M in PBP2	This work
PAO1 $PBP2_{V516M}$ ΔoprM	PAO1 carrying mutation V516M in PBP2 Δ*oprM::lox*	This work
PAO1 $PBP2_{V516M}$ ΔmexR	PAO1 carrying mutation V516M in PBP2 Δ*mexR::lox*	This work
PAO1 $PBP2_{V516M}$ ΔdacB	PAO1 carrying mutation V516M in PBP2 Δ*dacB::lox*	This work
PAO1 $PBP2_{V516M}$ ΔmexZ	PAO1 carrying mutation V516M in PBP2 Δ*mexZ::lox*	This work
E. coli		
OP50		Brenner S 1974
XL-1 blue	F'::Tn10 *proA*$^+$*B*$^+$ *lac1*^{q}Δ(*lacZ*)M15 *recA1 endA1 gyrA96* (*Nal*^{R}) *thi hsdR17* ($r_k^-m_k^-$) *mcrB1*	Laboratory collection
S17.1 λ-pir	RecA pro (RP4-2Tet::Mu Kan::Tn7)	Laboratory collection
S17 pEX_oprM::Gm	S17.1 λ-pir strain with pEX100Tlink containing 5'-3' flanking sequence of *oprM*::Gm*lox*	Mulet X et al 2011
S17 pEX_mexR::Gm	S17.1 λ-pir strain with pEX100Tlink containing 5'-3' flanking sequence of *mexR*::Gm*lox*	Mulet X et al 2011
S17 pEX_dacB::Gm	S17.1 λ-pir strain with pEX100Tlink containing 5'-3' flanking sequence of *dacB*::Gm*lox*	Moya B et al 2009
S17 pEX_mexZ::Gm	S17.1 λ-pir strain with pEX100Tlink containing 5'-3' flanking sequence of *mexZ*::Gm*lox*	Torrens G et al 2016
Plasmids		
pEX18Gm^R	Gm^R; *oriT*$^+$ *sacB*$^+$, gene replacement vector with MCS from pUC18	Hoang TT et al 1998
pEX_$PBP2_{V516M}$	pEX18Gm^R containing cloned region of PBP2 with V516M mutation	This work
pCM157	Tc^R, *cre* expression vector	Quénée L et al 2005

Table S2. Primers used in this work

**Primer**	**Sequence (5'-3')[a]**	**Use**	**Reference or source**
PBP2_F_EcoRI	TCGAATTCCACCGTGAATCTCATGGTCG	PBP2 mutagenesis	This work
PBP2_R_HindIII	TCAAGCTTCTGATCACCATGTGCTTGAG		
OprM_F_EcoRI	TCGAATTCGATCGGTACCGGCGTGATC	OprM inactivation	Mulet X et al 2011
OprM_R_BamHI	TCGGATCCAAGCCTGGGGATCTTCCTTC		
MexR_F_EcoRI	TCGAATTCAGCAGGGCCGGAACCAGTA	MexR inactivation	Mulet X et al 2011
MexR_R_BamHI	TCGGATCCAGAAGAACCCGTCGGCCGA		
DacB_F_EcoRI	TCGAATTCCGACCATTCGGCGATATGAC	DacB inactivation	Moya B et al 2009
DacB_R_BamHI	TCGGATCCCGCGTAATCCGAAGATCCATC		
MexZ_F_EcoRI	TCGAATTCATGCCGGCGACGCGCGCC	MexZ inactivation	Torrens G et al 2016
MexZ_R_BamHI	TCGGATCCTGATGGCGGACGATTGACGC		
PBP2_F_rtPCR	ACACGCTGGTGGAGATCCTC	Quantification of *pbpA* mRNA	This work
PBP2_R_rtPCR	TTCAGTTCCTGCTCGTTGATC		
RpsL-1	GCTGCAAAACTGCCCGCAACG	Quantification of *rpsL* mRNA	Oh H et al 2003
RpsL-2	ACCCGAGGTGTCCAGCGAACC		

[a]Sites for restriction endonucleases are underlined.

Brenner S. 1974. The genetics of *Caenorhabditis elegans*. *Genetics* 77:71–94. https://doi.org/10.1093/genetics/77.1.71

Hoang TT, Karkhoff-Schweizer RR, Kutchma AJ, Schweizer HP. 1998. A broad-host-range Flp-FRT recombination system for site-specific excision of chromosomally-located DNA sequences: application for isolation of unmarked *Pseudomonas aeruginosa* mutants. *Gene* 212:77–86. https://doi.org/10.1016/s0378-1119(98)00130-9

Moya B, Dötsch A, Juan C, Blázquez J, Zamorano L, Haussler S, Oliver A. 2009. Beta-lactam resistance response triggered by inactivation of a nonessential penicillin-binding protein. *PLoS Patho* 5:e1000353. https://doi.org/10.1371/journal.ppat.1000353

Mulet X, Moyá B, Juan C, Macià MD, Pérez JL, Blázquez J, Oliver A. 2011. Antagonistic interactions of *Pseudomonas aeruginosa* antibiotic resistance mechanisms in planktonic but not biofilm growth. *Antimicrob Agents Chemother* 55:4560–4568. https://doi.org/10.1128/AAC.00519-11

Oh H, Stenhoff J, Jalal S, Wretlind B. 2003. Role of efflux pumps and mutations in genes for topoisomerases II and IV in fluoroquinolone-resistant *Pseudomonas aeruginosa* strains. *MicrobDrug Resist* 9:323–328. https://doi.org/10.1089/107662903322762743

Quénée L, Lamotte D, Polack B. 2005. Combined sacB-based negative selection and cre-lox antibiotic marker recycling for efficient gene deletion in *Pseudomonas aeruginosa*. *Biotechniques*, 38:63–67. https://doi.org/10.2144/05381ST01

Torrens G, Cabot G, Ocampo-Sosa AA, Conejo MC, Zamorano L, Navarro F, Pascual Á, Martínez-Martínez L, Oliver A. 2016. Activity of ceftazidime-avibactam against clinical and isogenic laboratory *Pseudomonas aeruginosa* isolates expressing combinations of most relevant β-lactam resistance mechanisms. *Antimicrob Agents Chemother* 60:6407–6410. https://doi.org/10.1128/AAC.01282-16
